# Incomplete Selective Sweeps of *Microcystis* Population Detected by the Leader-End CRISPR Fragment Analysis in a Natural Pond

**DOI:** 10.3389/fmicb.2018.00425

**Published:** 2018-03-08

**Authors:** Shigeko Kimura, Mika Uehara, Daichi Morimoto, Momoko Yamanaka, Yoshihiko Sako, Takashi Yoshida

**Affiliations:** ^1^Graduate School of Agriculture, Kyoto University, Kyoto, Japan; ^2^School of Environmental Science, The University of Shiga Prefecture, Hikone, Japan

**Keywords:** *Microcystis aeruginosa*, CRISPR, spacer diversity, incomplete selective sweep, bottle neck, founder effect

## Abstract

The freshwater cyanobacterium *Microcystis aeruginosa* frequently forms toxic massive blooms and exists in an arms race with its infectious phages in aquatic natural environments, and as a result, has evolved extremely diverse and elaborate antiviral defense systems, including the clustered regularly interspaced short palindromic repeats (CRISPR)-CRISPR-associated genes (Cas) system. Here, to assess *Microcystis* population dynamics associated with exogenous mobile genetic elements such as phages and plasmids, we examined the temporal variation in CRISPR genotypes (CTs) by analyzing spacer sequences detected in a natural pond between June and October 2013 when a cyanobacterial bloom occurred. A total of 463,954 high-quality leader-end CRISPR sequences were obtained and the sequences containing spacers were classified into 31 previously reported CTs and 68 new CTs based on the shared order of the leader-end spacers. CT19 was the most dominant genotype (32%) among the 16 most common CTs, followed by CT52 (14%) and CT58 (9%). Spacer repertoires of CT19 showed mainly two different types; CT19_origin_, which was identical to the CT19 spacer repertoire of previously isolated strains, and CT19_new+_, which contained a new spacer at the leader-end of the CRISPR region of CT19_origin_, which were present in almost equal abundance, accounting for up to 99.94% of CT19 sequences. Surprisingly, we observed the spacer repertoires of the second to tenth spacers of CT19_origin_ at the most leader-end of proto-genotype sequences of CT19_origin_. These were observed during the sampling in this study and our previous study at the same ecosystem in 2010 and 2011, suggesting these CTs persisted from 2011 to 2013 in spite of phage pressure. The leader-end variants were observed in other CT genotypes. These findings indicated an incomplete selective sweep of *Microcystis* populations. We explained the phenomenon as follow; the abundance of *Microcystis* varied seasonally and drastically, resulting that *Microcystis* populations experience a bottleneck once a year, and thereby founder effects following a bottleneck mean that older CTs have an equal chance of increasing in prevalence as the CTs generated following acquisition of newer spacers.

## Introduction

Viruses infecting bacteria (phages) are ubiquitous and abundant in aquatic natural environments, with bacterial host-phage interactions shaping aquatic ecosystems (Suttle, 2005, 2007). Bacteria exist in an arms race with the highly abundant phages (Stern and Sorek, 2011; Avrani et al., 2012), and as a result, have evolved extremely diverse and elaborate antiviral defense systems, including: (a) resistance based on variation of virus receptors, (b) immunity [e.g., the restriction-modification system and the clustered regularly interspaced short palindromic repeats (CRISPR)-CRISPR-associated genes (Cas) system], and (c) dormancy induction and programmed cell death (e.g., the toxin-antitoxin and abortive infection systems) (Koonin et al., 2017).

CRISPR-Cas systems are the bacterial adaptive immune response (Barrangou et al., 2007). According to previous analyses based primarily on metagenomic sequences from natural environments and whole genome sequences from cultivated microorganisms, CRISPR-Cas systems have been found in the genomes of ∼10–80% of archaea and 10–40% of bacteria (Makarova et al., 2015; Burstein et al., 2016). They are composed of CRISPR loci, which are short direct repeats separated by non-repetitive sequences (spacers), and CRISPR-associated genes (Barrangou et al., 2007; Sorek et al., 2008). CRISPR-Cas systems can incorporate unique fragments of DNA from invading mobile genetic elements, such as phages and plasmids. The transcripts of these unique spacers are then used to target the cognate sequences in viral or plasmid genomes, thereby protecting bacteria and archaea from viral infection and plasmid transfer. Generally, it seems that spacers are acquired at the leader-end of the CRISPR loci, with the older spacers remaining at the trailer end (e.g., *Escherichia coli, Streptococcus thermophiles, Streptococcus agalactiae*) (Barrangou et al., 2007; Andersson and Banfield, 2008; Stern and Sorek, 2011; Lopez-Sanchez et al., 2012; Swarts et al., 2012). However, there is an exception; acquisition of new spacers internal to a CRISPR array was observed in wet laboratory experiments in *Sulfolobus solfataricus* (Erdmann and Garrett, 2012). Although the manner of acquisition of spacer is dependent on host microorganisms, lots of CRISPR-based typing studies were performed based on the idea that CRISPR loci also provide a chronological record of infections, with the most recent infection represented by the most leader-end spacer (Barrangou and Dudley, 2016). This historical record of infection can provide insights into past phage–bacterial interactions in natural ecosystems, with potential for resolution in both space and time.

Several recent studies have used CRISPR-based typing to distinguish closely related strains and co-evolutionary dynamics between phage and bacteria that have shaped the species composition within bacterial populations, contributing to events such as sweeps and bottlenecks. These studies have examined phage–bacterial relationships in several environments, including acid mine drainage biofilms (Andersson and Banfield, 2008; Tyson and Banfield, 2008), microbial mats in hyperthermophilic environments (Heidelberg et al., 2009; Held et al., 2010, 2013), industrial fermentation set-ups (Paez-Espino et al., 2013; Briner and Barrangou, 2014), the human oral cavity (Pride et al., 2011), and human and animal guts (Touchon and Rocha, 2010; Touchon et al., 2011).

In two closely related strains of *Leptospirillum* group II, one of the dominant species in biofilms formed during acid mine drainage, spacers at the trailer-end of the CRISPR locus were generally in a conserved order (with some spacer loss) in both strains, while spacers in the middle of the locus were population specific. Toward the leader end of the locus, the spacers became strain specific, suggesting that the populations had experienced a selective sweep for 5 months (Andersson and Banfield, 2008; Tyson and Banfield, 2008). In contrast, diversity of the spacer repertoires of CRISPR loci in *Sulfolobus* population coexisted and many of the spacer repertoires were conserved, showing no evidence of a selective sweep (Held et al., 2010). Thus, the coevolution dynamics of CRISPR loci differs depending on the interaction between bacteria and their phage.

*Microcystis aeruginosa* forms toxic cyanobacterial blooms throughout the world (Guo, 2007; Paerl and Huisman, 2008). A comparative genomic study showed that the *M. aeruginosa* NIES-843 genome contained remarkably abundant and diverse antiviral defense genes, including a CRISPR-Cas system (Makarova et al., 2012, 2013), suggesting strong co-evolutionary interactions with diverse active phages (Kuno et al., 2012). Therefore, *Microcystis* appears to be an ideal subject to study host-phage co-evolution in natural aquatic environments (Kuno et al., 2014). CRISPR-Cas subtype I-A, III-A, III-B, and I-D systems have so far been identified in *Microcystis* genomes (Yang et al., 2015). Subtype I-D CRISPR-Cas2 was found in 12 of the 14 sequenced *Microcystis* genomes, and of the 84 spacers matched to known phages and plasmids, 56 were from subtype I-D CRISPRs, suggesting that the subtype I-D CRISPR system is an influential defense system for this species (Kuno et al., 2012; Yang et al., 2015). Analysis of the leader-end of the subtype I-D CRISPR system in a limited number of *Microcystis* isolates revealed that multiple different CRISPR genotypes coexist within a small pond, and that some genotypes were present in all samples throughout a 2-year sampling period (Kuno et al., 2014). This findings suggest that the *Microcystis* population has not experienced selective sweep (Kuno et al., 2012, 2014). To assess this issue, we examined the temporal variation in CRISPR genotypes of *Microcystis* population by analyzing spacer sequences detected in a natural pond between June and October 2013, during a cyanobacterial bloom, using next-generation sequencing (NGS) technology.

## Materials and Methods

### Study Site and Sampling Method

Surface water samples were collected once or twice per month from June to October 2013 during a cyanobacterial bloom from Hirosawanoike Pond (35°026′ N, 135°690′ E) in central Kyoto, Japan. Pond water was stored in a brown bottle and transported to the laboratory within 1 h. For DNA extraction, 100 mL of pond water were sonicated gently and then cells were harvested using centrifugation at 1,680 × *g* for 10 min (Yoshida et al., 2003). The resulting pellet was stored at -20°C until DNA analysis.

### DNA Extraction, CRISPR Amplification, and MiSeq Amplicon Sequencing

DNA extraction was performed using the xanthogenate method as described previously (Yoshida et al., 2003), and purified DNA was resuspended in 30 μL of deionized water. The amount and purity of the extracted DNA were determined using optical density comparison at 260 and 280 nm. Each DNA extract was used as a template for polymerase chain reaction (PCR) analysis to determine CRISPR arrays.

CRISPR amplicon sequencing was performed using two-step PCR amplification, followed by sequencing using an Illumina MiSeq amplicon sequencing protocol^1^. First-round amplification of the leader-end fragment of the CRISPR loci was performed using CRISPR-specific primers MaeCRf2 (5′-CTTATCCCGTAAGGTTTTGC-3′) and MaeCRrGT (5′-GGTTTAAGATTAATTGGAACGT-3′) or MaeCRrCA (5′-GGTTTAAGATTAATTGGAACCA-3′). MaeCRf2 primer was designed based on leader sequence and MaeCRrGT and MaeCRrCA were based on CRISPR repeat as described by Kuno et al. (2012). Adapter overhang sequences TCGTCGGCAGCGTCAGATGTGTATAAGAGACAG and GTCTCGTGGGCTCGGAGATGTGTATAAGAGACAG were added to the 5′ ends of the forward and reverse primers, respectively. PCR amplification was performed using a PCR Thermal Cycler Dice Touch apparatus (TaKaRa Bio). Each 25-μL reaction mixture contained KAPA HiFi HotStart ReadyMix (KAPA BIOSYSTEMS) and 75 ng of DNA extracted from the environmental water samples. The reaction conditions were as follows: an initial denaturation at 95°C for 3 min, followed by 25 cycles of 95°C for 30 s, 60°C for 30 s, and 72°C for 30 s, with a final extension at 72°C for 5 min. Amplicons were then purified using Agencourt AMPure XP Beads (Beckman Coulter) according to the manufacturer’s instructions.

For the second-round PCR amplification, 5 μL of product from the first round of PCR were used as template, and 5 μL of each Nextera XT index (N50X and N70X) were added to the PCR reaction mixture containing 1× KAPA HiFi HotStart ReadyMix (KAPA BIOSYSTEMS). Each sample had a different combination of the N50X and N70X indexes. The reaction conditions were as follows: an initial denaturation at 95°C for 3 min, followed by 8 cycles of 95°C for 30 s, 55°C for 30 s, and 72°C for 30 s, with a final extension at 72°C for 5 min. Product obtained from the second-round PCR was cleaned using Agencourt AMPure XP beads (Beckman Coulter). Sample DNA concentration was determined using a Qubit Fluorometer (Thermo Fisher Scientific), and the mean length of each product was determined using an Agilent Bioanalyzer HS DNA Kit (Agilent). Samples were then diluted to 8 nM DNA with 10 mM Tris buffer and pooled. The amplicon library was combined with 10% PhiX control library and sequenced using the Illumina Miseq system (Illumina) with a v3 600-cycle (300 cycles per read) paired end kit.

### CRISPR Spacer Analysis

Sequencing raw reads from different samples were identified and separated according to specific barcodes, and adapter sequences were automatically trimmed by the Illumina Miseq system. The quality of the raw fastq files (R1 for forward and R2 for reverse files) was checked using FastQC^2^. Trimmomatic v0.33 (Bolger et al., 2014) was used to remove ambiguous and low quality sequence reads by scanning the reads with a 4-base-wide sliding window. Threshold values of the average quality score per base were set at 10 for R1 sequences and 20 for R2 sequences, respectively. In this sequencing, qualities of the R2 reads were very low due to a developing Illumina v3 version. When we merged these filtered sequences, the highest number of merged sequences were generated. Additionally, reads shorter than 200 bp (R1 sequences) and 70 bp (R2 sequences) were dropped. Sequence reads were then quality filtered using the FASTX-Toolkit^3^. The quality filtering settings were a minimum quality score of 20 over at least 80% of the sequence read. The high-quality paired-end reads obtained after trimming and quality filtering were merged using FLASH^4^, with a 60-bp minimum overlap parameter, 301-bp maximum overlap parameter, and 90% identity between R1 and R2 sequence reads. To extract spacers using CRISPRtionary (Grissa et al., 2007), the high-quality CRISPR amplicon sequences were grouped using CD-HIT-EST (Li and Godzik, 2006), with parameters of 90% length overlap and 95% identity. The identity was optimized by a pre-test in which we clustered the CRISPR sequences with identities from 90 to 98%. CRISPRtionary was used to extract spacers between repeats based on repeat sequences from representative sequences of each group clustered by CD-HIT-EST. Each spacer was annotated with a sequential number on the basis of sequence identity. Considering sequencing errors, up to six mismatches between spacers were allowed when searching identical spacers. Based on the extracted and annotated spacers, each CRISPR amplicon sequence was classified into a specific CRISPR type (CT; CT1–CT35), as defined by Kuno et al. (2012, 2014), based on the shared order of the leader-end spacers. Representative CRISPR amplicon sequences with no spacers in common with types CT1–CT35 were classified as new CRISPR genotypes. A new CRISPR genotype was defined using CRISPR sequences with more than three new spacers. No spacer was shared among these assigned CTs. For amplicons with only one spacer, if these spacers were shared with those of the CTs, we assigned them into the CTs. Putative origins of spacers were identified by nucleotide similarity search against the NCBI non-redundant database using Blastn (Camacho et al., 2009) with an *E*-value threshold of 10^-5^.

### Diversity Analysis

The Shannon and Chao1 indexes were estimated for the obtained sequences using PAST v2.09 (Hammer et al., 2008) and EstimateS v8.2.0 (Colwell, 2006), respectively. Rarefaction curves were obtained for each sample using PAST v2.09.

### Real-Time PCR Amplification

To quantify the total abundance of CT19 and CT29 in the *Microcystis* bloom, a real-time PCR assay was performed using primers targeting the phycocyanin intergenic spacer (PC-IGS) locus (Yoshida et al., 2010), CT19-specific spacers (Spacer ID: 64 and 66), and CT29-specific spacers (Spacer ID: 89 and 156) (**Supplementary Table S3**) (Kuno et al., 2012). Three replicates were used to quantify gene copy numbers. The copy numbers of each product were quantified using SYBR Premix Ex Taq (Tli RNaseH Plus; TaKaRa Bio) with 1 μL of environmental DNA. Individual assays were performed for each primer set using the following cycle parameters: for total *Microcystis* (PC-IGS), denaturation at 95°C for 15 s, annealing at 60°C for 15 s, and extension at 72°C for 30 s; and for CT19 and CT29, denaturation at 95°C for 15 s, annealing at 65°C for 15 s, and extension at 72°C for 30 s. Simultaneously, we constructed a standard curve using ten-fold serially diluted DNA containing the PCR product (10^5^–10^7^ DNA copy numbers) from a *Microcystis* isolate and calculated these gene numbers in environmental samples using a standard curve.

### Nucleotide Sequences

The CRISPR amplicon sequencing data were deposited in the DDBJ Sequence Read Archive (DRA) under accession numbers DRR106729–DRR106735.

## Results

### Analysis of CRISPR Spacer Sequences

In total, 474,002 sequences were obtained from seven samples by paired-end sequencing (**Supplementary Table S1**). After trimming, filtering, and merging these sequences, 463,954 high-quality sequences were selected, with 3,199–97,203 sequences per sample (**Supplementary Table S1**). We extracted a total of 676,460 spacers from the amplicon sequences based on the detection of the *Microcystis* direct repeats (Kuno et al., 2012) using CRISPRtionary (Grissa et al., 2007), of which 713 were unique. Overall, each amplicon sequence contained between one and five spacers, with one, two, and more than three spacers found in 62, 27, and 8% of amplicons, respectively (**Supplementary Table S1**). Two percent of amplicons contained no spacers or undetectable spacers because the repeat sequences could not be detected. The sequences containing spacers were classified into 31 previously reported CRISPR genotypes (Kuno et al., 2014) and 68 new CRISPR genotypes (CT36 to CT103), which only included sequences containing more than three novel spacers in this study (**Supplementary Table S3**). We discarded amplicon sequences containing only one or two spacers defined as novel (0.2% of total amplicon sequences included one or two spacers) because these sequences were not able to be classified into any CRISPR genotypes. However, the remaining 98.8% of amplicons could be classified. Of the total amplicon sequences, 0.06% were singletons, and these sequences were also discarded because we could not rule out sequencing errors.

We found 11 proto-spacers among 398 spacers tested (**Supplementary Table S3**), including *Microcystis* cyanophage Ma-LMM01, MaMV-DC and small cryptic plasmids (PMA1, pMA1 and pMA2) although most of spacers at the leader-end had unknown origins. We identified *Microcystis* cyanophage Ma-LMM01- and MaMV-DC-derived spacers at the most leader-end in CT15 and CT18 (**Table 1** and **Supplementary Figure S3**). Other identified spacers could be attributed to *Microcystis* plasmids PMA1, pMA1 and pMA2. In marine *Synechococcus* metagenomes, similar small cryptic plasmids were found and hypothesized to facilitate horizontal gene transfer and phage resistance (Palenik et al., 2009; Ma et al., 2012). Small cryptic plasmids in *Microcystis* populations may facilitate host genetic diversity via horizontal gene transfer or chromosomal integration.

**Table 1:** Spacers showing sequence identity to foreign genetic element.

CRISPR type	Spacer name	Spacer ID	Foreing genetic element	Identity^∗^
CT5	CT5:1	386	Plasmid pMA1	30/30
CT15	CT15:-1	792	*Microcystis* phage MaMV-DC	38/38
CT18	CT18:-2	771	*Microcystis* phage Ma-LMM01 and MaMV-DC	35/35
CT24	CT24:16	479	Plasmid PMA1	32/32
CT29	CT29:21	172	Plasmid pMA1	36/36
CT36	CT36:1	511	Plasmid pMA2	38/38
CT45	CT45:4	832	Plasmid pMA2	37/37
CT55	CT55:2	583	Plasmid PMA1	37/38
CT76	CT76:1	678	Plasmid PMA1	35/36
CT82	CT82:1	713	Plasmid pMA1	34/34
CT82	CT82:2	714	Plasmid pMA1	34/34

*^∗^Nucleotide sequence identity is indicated as the number of identical bacterial bases/total bases of a spacer.*

All rarefaction curves which present the relationship between number of sequences and richness in CRISPR genotypes, except for the curve generated for the sample from June, reached plateaus (**Supplementary Figure S1**). In addition, the Chao1 indexes were comparable with the number of CRISPR genotypes in the samples (**Supplementary Table S2**). Although there is a smaller number of sequences in June compared to other sampling day, Chao1 index showed 19 in Jun 11th sample which is comparable to 18 CTs observed, suggesting the sequence number was sufficient to analyze the data. These findings suggested that there were CRISPR amplicon sequences sufficient to a CRISPR analysis in each sample.

### Dominant CRISPR Genotypes and Real-Time PCR of CT19 and CT29

**FIGURE 1:**
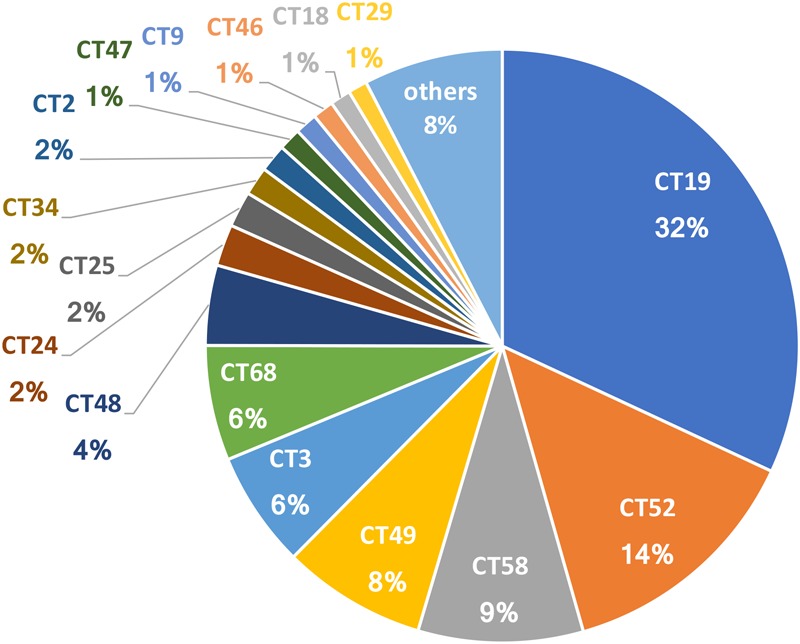
The abundance of sequences for each CRISPR genotype relative to the total number of sequences in the samples collected from Hirosawanoike Pond from June–October 2013. Sixteen CRISPR genotypes accounted for up to 92% of the total number of sequences obtained. The remaining sequences belonged to the 83 rare CRISPR genotypes.

**FIGURE 2:**
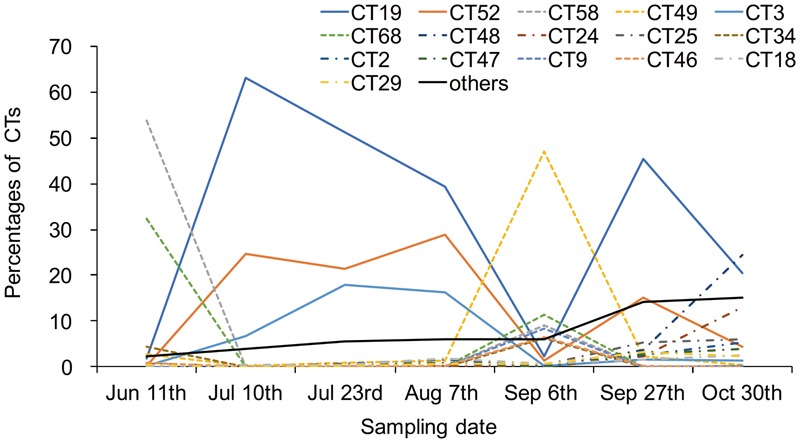
Temporal dynamics of the number of sequences for each of the 16 dominant CRISPR genotypes relative to the total numbers of sequences in the samples collected from Hirosawanoike Pond from June–October 2013.

Of the 99 CTs detected in this study, 16 CTs accounted for up to 92% and each of them included more than 1% of all CRISPR amplicon sequences. CT19 was the most dominant genotype (32%) among the 16 most common CTs, followed by CT52 (14%) and CT58 (9%) (**Figure 1**). The remaining 83 less common CTs accounted for only 8% of all CRISPR amplicon sequences (rare CRISPR genotypes) (**Figure 1**). The 15 dominant CTs coexisted during the sampling period, and their individual dominance trajectories could be distinguished into three patterns: (1) CT19, CT52, and CT3; (2) CT9, CT34, CT46, CT49, CT58, and CT68; and (3) CT2, CT18, CT24, CT25, CT29, CT47 and CT48 (**Figure 2**).

A previous study showed that CT29 was the dominant genotype in the sampling pond from 2010 to 2011 (Kuno et al., 2014); however, we only detected a prevalence of 1% for this particular genotype. To validate our data, we performed real-time PCR using CT19- and CT29-specific primers. The copy numbers of the *Microcystis* PC-IGS gene ranged from 1.3 × 10^5^ to 1.8 × 10^6^ copies/mL between June and October 2013 (**Supplementary Figure S2A**). Nine copies of CT19 were detected in June, and then peaked at 3.8 × 10^4^ copies/mL in July and 5.5 × 10^4^ copies/mL in August, before decreasing to 4.9 × 10^2^ copies/mL at the beginning of September (6th September). Copy numbers peaked again, to 3.2 × 10^5^ copies/mL, at the end of September (27th September) (**Supplementary Figure S2A**). The CT29 copy numbers were below the detection limit between June and mid-July (10th July), but gradually increased to 2.1 × 10^3^ copies/mL by the end of September (27th September).

The ratios of the total copy numbers of CT19 and CT29 to that of PC-IGS were 34 and 0.2% (**Supplementary Figure S2A**), respectively, which showed similar trends to the observed copy numbers (**Supplementary Figure S2B**). Based on MiSeq sequencing data, the relative abundance of CT19 and CT29 sequences was 32 and 1% of the total number of sequences (**Figure 1**), respectively, which agreed with the real-time PCR data (**Supplementary Figure S2B**). These findings confirmed that our CRISPR leader-end analysis based on NGS reflected the relative numbers of CTs in the natural population.

### Spacer Repertoires of CRISPR Genotypes

**FIGURE 3:**
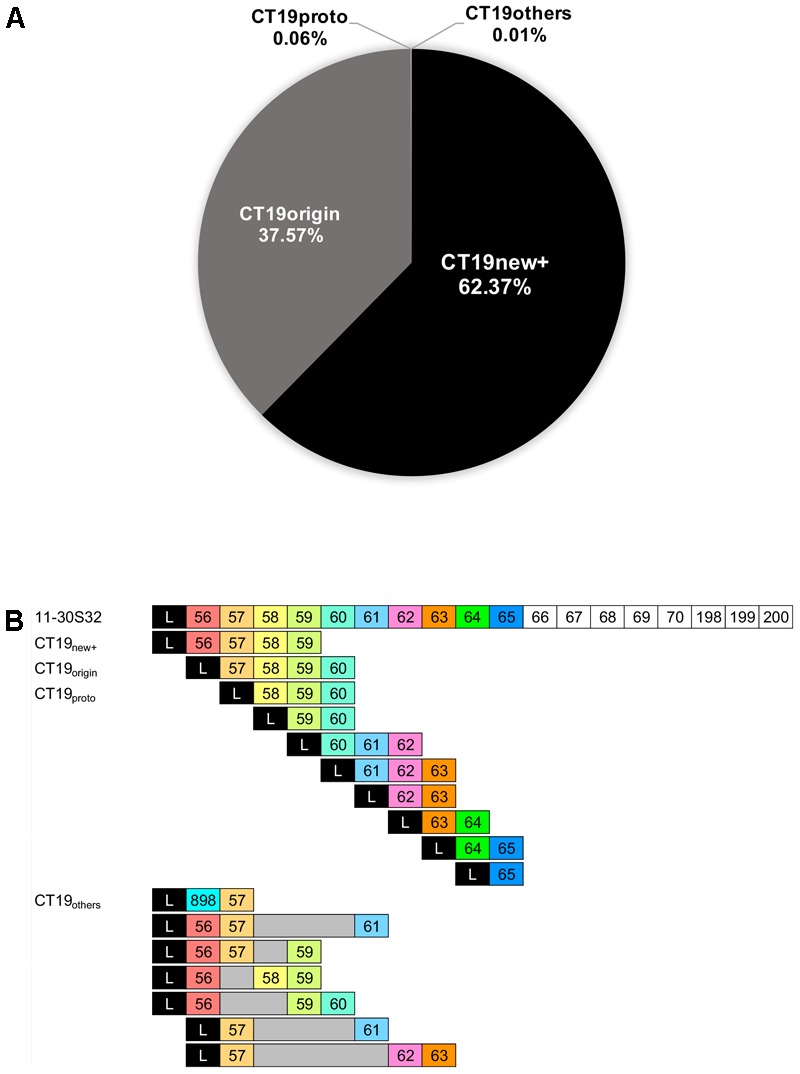
The abundance of each CT19 variants (CT19_origin_, CT19_new+_, CT19_proto_, and CT19_others_) relative to the total number of CT19 sequences **(A)**, and schematic representation of spacer repertoires of CT19 sequences obtained from Hirosawanoike Pond from June–October 2013 **(B)**. Names of an isolate and CT19 variants are indicated on the left. 11-30S32 was an isolate from Hirosawanoike Pond by Kuno et al. (2014). In the first line, a spacer repertoire of 11-30S32 was indicated. Each box indicates a spacer, and the number in each box indicates a spacer ID. L indicates a leader sequence. Gray boxes indicate a deletion of spacers.

Eleven dominant CTs (CT2, CT3, CT18, CT19, CT24, CT25, CT29, CT47, CT48, CT49, and CT52) and nine rare CTs (CT7, CT11, CT15, CT16, CT26, CT33 CT53, CT54, and CT83) showed variations in their spacer repertoires at the leader-end. The most dominant genotype CT19 consisted of two main variants accounting for up to 99.94% of sequences (**Figure 3A**). One of the variants was identical to the CT19 CRISPR repertoire of previously isolated strains (CT19_origin_) (Kuno et al., 2014), while the other contained a new spacer at the leader-end of the CRISPR region of CT19 (CT19_new+_) (**Figure 3B** and **Table 2**). Only 0.06% of the CT19 sequences showed a proto-genotype of CT19 (CT19_proto_) (**Figure 3A**). The CT19_proto_ sequences contained the 3rd to 10th spacers of an isolate 11-30S32 (Kuno et al., 2014) assigned as CT19_new+_ at the most leader-end (**Figure 3B**). Up to 0.01% of the CT19 sequences included other types (CT_others_) of variants of CT19_origin_, which missed spacers found in CT19_origin_ and acquired a new spacer found in CT19_proto_ (**Figure 3B** and **Table 2**).

**Table 2:** Definition of CT variants. Same number and color in a box indicates same spacer.

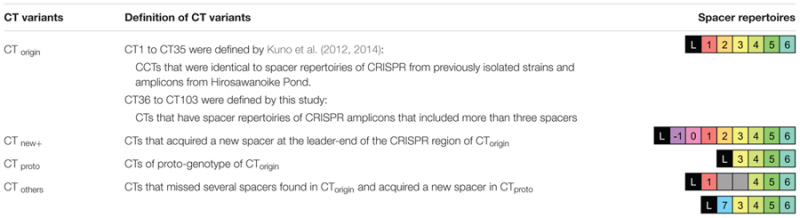

**FIGURE 4:**
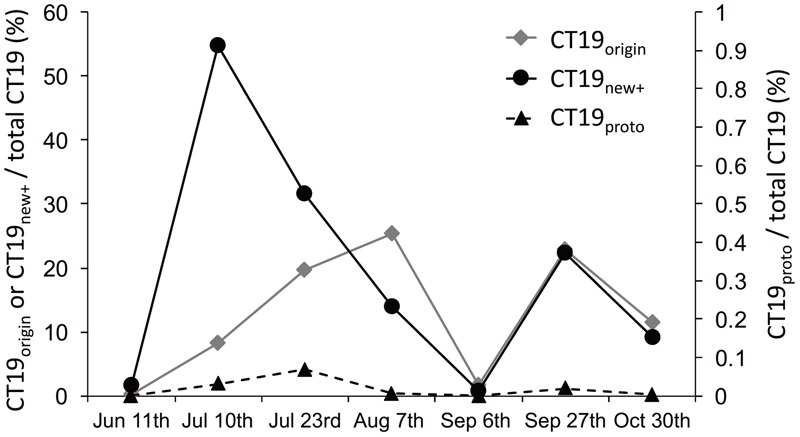
Temporal dynamics in the prevalence of CT19_origin_, CT19_new+_ (left *Y*-axis), and CT19_proto_ (*Y*-axis) relative to the total abundance of CT19 genotypes in samples collected from Hirosawanoike Pond from June–October 2013.

The relative frequency of CT19_new+_, CT19_origin_, and CT19_proto_ oscillated during the sampling period (**Figure 4**). CT19_new+_ showed peak prevalence in mid-July (10th July), accounting for 55% of the CT19 sequences, then decreased to 0.7% of sequences, before rising again to 22% of CT19 sequences. The prevalence of CT19_origin_ increased gradually up to 25% of sequences by 7th August, then decreased to 1.6% of CT19 sequences on 6th September. This variant subsequently increased again to 23%. The prevalence of CT19_proto_ showed between 0 and 0.068% of the CT19 sequences during sampling period.

We next subdivided each of the CT genotypes in the same manner as for CT19 based on the sequences of CT_origin_ isolates (CT1 and CT35) (Kuno et al., 2012, 2014). The amplicon sequences with the largest numbers of spacers (CT36 to CT103) were used as CT_origin_. As such, CT_new+_ indicated CRISPR genotypes to which a new spacer was added at the leader-end of CT_origin_. CT_new+_ genotypes were found in two dominant (CT18 and CT29) and eight rare (CT7, CT11, CT15, CT16, CT33, CT53, CT54, and CT83) CTs (**Supplementary Figure S3**). CT_proto_ indicated CRISPR genotypes in which the leader-end spacers were older than the 2nd spacer of CT_origin_. CT_proto_ was found in 10 dominants (CT2, CT3, CT18, CT24, CT25, CT29, CT47, CT48, CT49, and CT52) and four rare (CT11, CT16, CT26, and CT33) CTs (**Supplementary Figure S3**). Further, CT_others_ genotypes that had acquired new spacers were found in CT11, CT33, and CT53 sequences (**Supplementary Figure S3**). Spacer deletion (CT_others_) was also shown to occur in some CT19, CT24, and CT48 sequences (**Figure 3B** and **Supplementary Figure S3**). These diverse spacer repertoires at the leader-end co-existed during the sampling period (**Supplementary Figure S4**).

## Discussion

We have no experimental evidence to show whether the *Microcystis* CRISPR system provides a phage defense. However, Kuno et al. (2012) found several spacers matching known foreign genetic elements for *Microcystis* in CRISPR loci of *Microcystis* strains. In addition, we identified two *Microcystis* cyanophage-derived spacers at the most leader-end in CT15_new+_ and CT18_new+_ in this study, and the CT15_origin_ and CT18_origin_ have been obtained from environmental DNA of the same pond, in 2010 (Kuno et al., 2012). These suggests that *Microcystis* CRISPR system had been functional at least in the past, and some CTs interacted with *Microcystis* cyanophages and the spacers might have been acquired recently. Thereby, the spacer repertoires represent a history of previous host-parasite interaction.

As shown by a pioneering analysis of CRISPR-phage interactions in an acid mine drainage ecosystem, transition from clonal to non-clonal loci in two closely related *Leptospirillum* group strains indicated a recent selective sweep of 5 months duration (Andersson and Banfield, 2008; Tyson and Banfield, 2008). Likewise, studies on *Streptococcus thermophiles* and *Lactobacillus buchneri*, which are key species exploited in the formulation of dairy culture systems for food industries, revealed population dynamics driven by a selective sweep, where active CRISPR loci can acquire novel spacers on a daily basis as a result of phage pressure (Paez-Espino et al., 2013; Briner and Barrangou, 2014). In these cases, the environment may be relatively stable (i.e., conductivity, pH and temperature in some sites of acid mine drainage and controlled temperature and nutrients in fermented industries), resulting in simplified microbial populations and host-phage coevolutionary dynamics. Our current observations of population dynamics based on CRISPR loci differed from these previous studies.

We previously observed the co-existence of diverse spacer repertoires at the leader-end of CRISPR loci in *Microcystis* isolates from Hirosawanoike Pond from three samplings conducted in the summer of 2010/2011, suggesting *Mycrocystis* populations have not experienced a selective sweep (Kuno et al., 2012, 2014). New generation sequencing technologies can be used to deeply characterize diversifying populations, thereby providing a powerful general method for high-resolution typing of CRISPR loci. In the current study, we used this technology to investigate the spacer repertoires at the leader-end of *Microcystis* CRISPR loci over a 5-month period. Here, we showed that the 16 dominant CTs coexisted and varied with trajectories distinguished into three patterns during the sampling period and 8 CTs of them were also found in 2010 and 2011 (**Figure 2**). The most prevalent CRISPR genotype, CT19, contained two variants, CT19_origin_ and CT19_new+_, which were present in almost equal abundance. CT19_origin_ was also observed in amplicons obtained in 2010, and CT19_new+_ in isolates collected in 2011 (Kuno et al., 2014), from the same ecosystem as examined in this study, suggesting these CTs persisted from 2011 to 2013 in spite of phage pressure. In our previous study, assemblages of Ma-LMM01 type*-Microcystis* phage were found to be composed of 11 genotypes based on sequences of a tail sheath protein. These genotypes coexisted and oscillated in the same sampling pond (Kimura et al., 2013). Thus, a negative frequency-dependent selection by phage infection as be referred to as constant-diversity dynamics may contribute to the dynamics of the dominant CTs.

Surprisingly, we observed that the spacer repertoires of the second to tenth spacers (spacer ID: 58–65) of CT19_origin_ at the most leader-end of CT19_proto_ sequences were present during the same sampling period. Additionally, our data indicated spacer variation in leader-end spacers in 11 dominant CTs and 9 rare CTs as was observed in CT19. These findings indicated that the *Microcystis* population has not experienced a complete selective sweep as was seen in populations of *Leptospirillum, Streptococcus*, and *Lactobacillus* (Andersson and Banfield, 2008; Tyson and Banfield, 2008; Paez-Espino et al., 2013; Briner and Barrangou, 2014). In contrast to the their habitats, environmental factors such as temperature and nutrient concentrations undergo seasonal changes in the *Microcystis* habitat (Paerl and Otten, 2013). As such, the abundance of *Microcystis* increases during cyanobacterial blooms in summer, but drastically decreases in winter (Harke et al., 2016). Also, *Microcystis* bloom usually occur from June to October every year in this study pond, while we cannot observe the bloom from December to May. Our previous study indicated that *Microcystis* abundance (according to PC-IGS gene copy numbers) varied seasonally in this pond, ranging from 1.3 × 10^3^ to 5.8 × 10^8^ copies mL^-1^ from April to November between 2006 and 2010 (Yoshida et al., 2010; Kimura et al., 2012, 2013). This means that *Microcystis* populations experience a bottleneck once a year in this pond, preventing a complete selective sweep. Founder effects following a bottleneck mean that older CTs have an equal chance of increasing in prevalence as the CTs generated following acquisition of newer spacers in the next blooming, resulting in an incomplete selective sweep of *Microcystis* populations.

*Microcystis* can form massive blooms in natural environments, making it easier for phage challenge to occur. However, in contradiction of the increasing phage challenge, *Microcystis* populations remain highly abundant in natural environments. This might be explained by the generation and maintenance of a diverse array of CRISPR loci within the *Microcystis* genomes, which act as a phage defense system.

## Author Contributions

SK prepared the draft manuscript. MU and MY performed the experiments. DM and YS contributed to manuscript discussion and revision. TY contributed to the experimental design, discussion of results, manuscript revision, and overall support of this study.

## Conflict of Interest Statement

The authors declare that the research was conducted in the absence of any commercial or financial relationships that could be construed as a potential conflict of interest.

## Footnotes

1 https://www.illumina.com/index-d.html

2 https://www.bioinformatics.babraham.ac.uk/projects/fastqc/

3 http://hannonlab.cshl.edu/fastx_toolkit/

4 http://ccb.jhu.edu/software/FLASH/

## Supplementary Material

FIGURE S1 Rarefaction curves for CRISPR genotypes in each sample collected from Hirosawanoike Pond from June–October 2013 generated using PAST software.

FIGURE S2 Temporal dynamics of the copy numbers of CT19, CT29, and total *Microcystis* (PC-IGS) genomes **(A)**, and abundance of CT19 and CT29 relative to total *Microcystis* **(B)**, in samples collected from Hirosawanoike Pond from June–October 2013. The CT19, CT29, and PC-IGS gene copy numbers per milliliter were determined by real-time polymerase chain reaction analysis. Data points are averages from three experiments, and error bars indicate standard deviations. Percentages were calculated by dividing the CT19 or CT29 copy numbers by the total *Microcystis* (PC-IGS) copy number.

FIGURE S3 Spacer repertoires of dominant and rare CRISPR genotypes in samples collected from Hirosawanoike Pond from June–October 2013. Names of an isolate and CT variants are indicated on the left. “10-xx and 11-xx” and “2010Env.” indicates names of CRISPR sequences obtained from isolates and environmental DNA, respectively, by Kuno et al. (2012, 2014). Each box indicates a spacer, and the number in each box indicates a spacer ID. L indicates a leader sequence. Gray boxes indicate a deletion of spacers.

FIGURE S4 Abundance (%) of CT_origin_, CT_new+_, and CT_proto_ in samples collected from Hirosawanoike Pond from June–October 2013.

TABLE S1 Sequencing information for each sample used in the analysis of CRISPR genotypes in Hirosawanoike Pond.

TABLE S2 Number of CRISPR genotypes and diversity index in each sample collected from Hirosawanoike Pond from June–October 2013.

TABLE S3 Spacer sequences found in this study and foreign genetic element with which the spacer shows sequence identity.
