# Supplementary material for: Incomplete Selective Sweeps of Microcystis Population Detected by the Leader-End CRISPR Fragment Analysis in a Natural Pond
Source: Front Microbiol. 2018 Mar 8;9:425. doi: 10.3389/fmicb.2018.00425 (PMC5852275; doi:10.3389/fmicb.2018.00425)
Supplement: FIGURE S1 — Rarefaction curves for CRISPR genotypes in each sample collected from Hirosawanoike Pond from June–October 2013 generated using PAST software. [file Image_1.PDF]

Figure S1

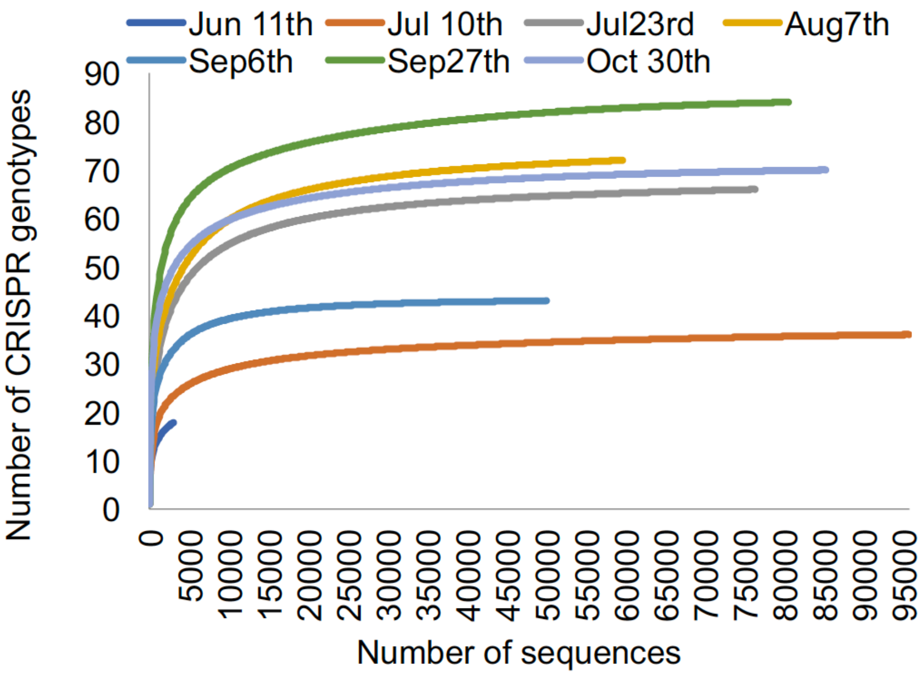
Jun 11th
Jul 10th
Jul23rd
Aug7th
Sep6th
Sep27th
Oct 30th
Number of CRISPR genotypes
0
10
20
30
40
50
60
70
80
90
0
5000
10000
15000
20000
25000
30000
35000
40000
45000
50000
55000
60000
65000
70000
75000
80000
85000
90000
95000
Number of sequences

Figure S2

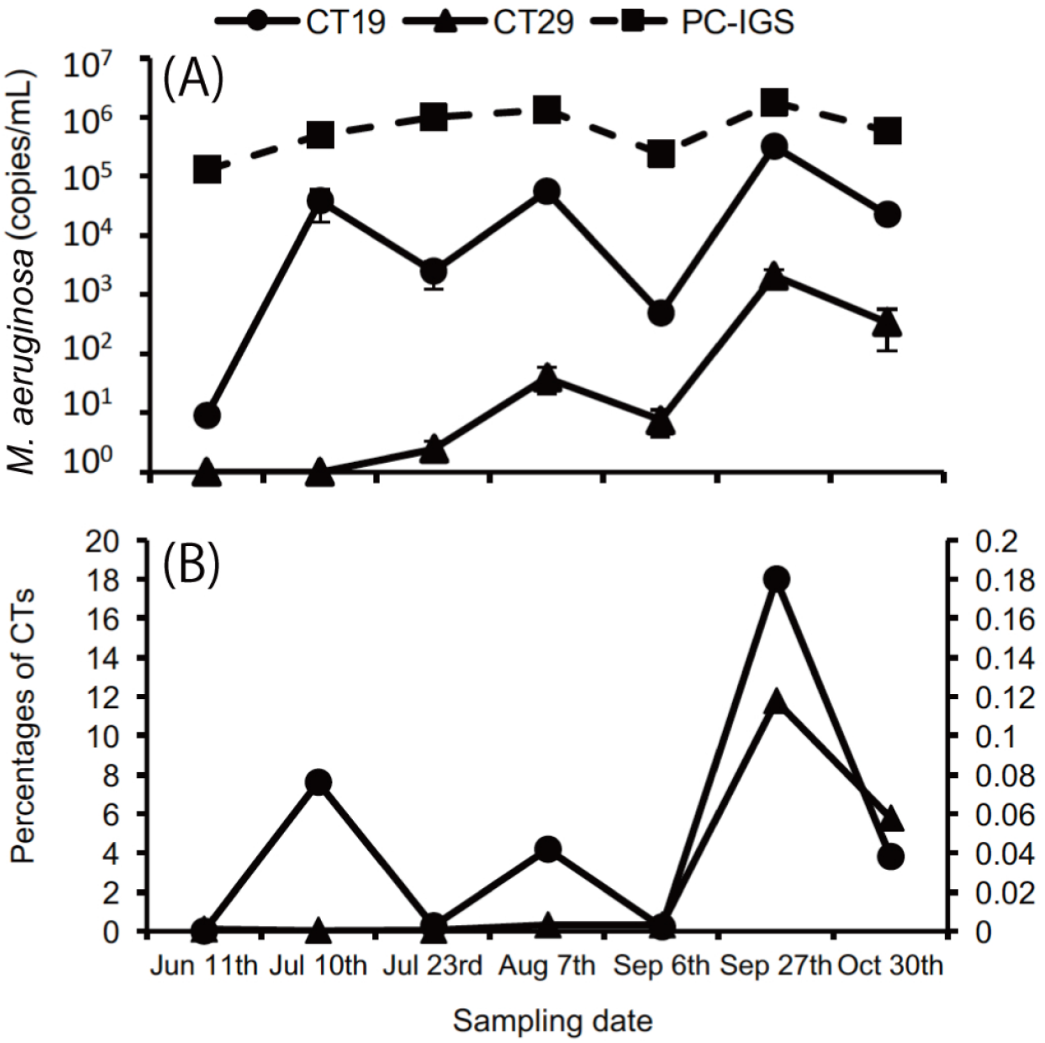

CT19
CT29
PC-IGS
(A)
10^7
10^6
10^5
10^4
10^3
10^2
10^1
10^0
M. aeruginosa (copies/mL)
(B)
Percentages of CTs
20
18
16
14
12
10
8
6
4
2
0
0.2
0.18
0.16
0.14
0.12
0.1
0.08
0.06
0.04
0.02
0
Jun 11th
Jul 10th
Jul 23rd
Aug 7th
Sep 6th
Sep 27th
Oct 30th
Sampling date

Figure S3.

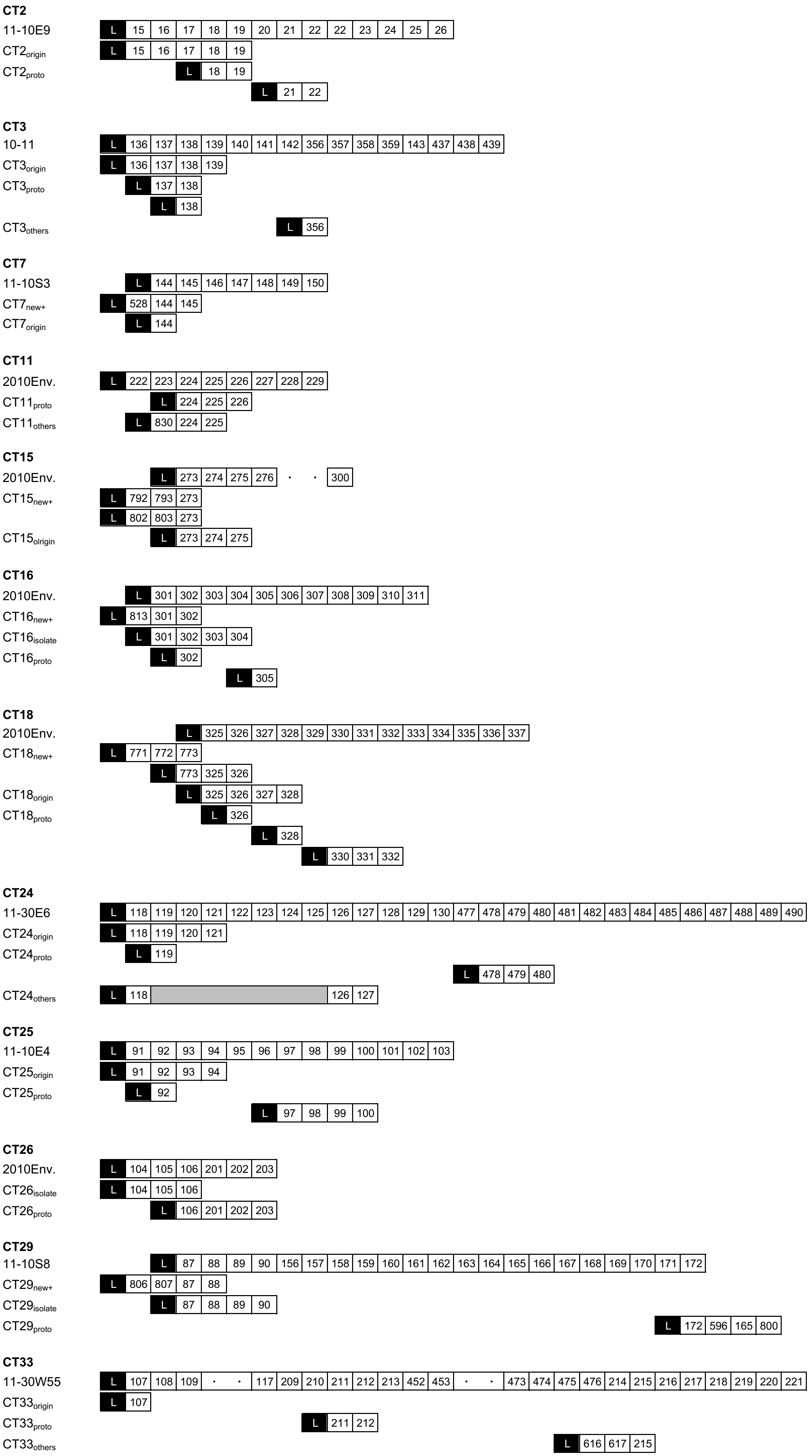

CT2
11-10E9 L 15 16 17 18 19 20 21 22 22 23 24 25 26
CT2origin L 15 16 17 18 19
CT2proto L 18 19
L 21 22
CT3
10-11 L 136 137 138 139 140 141 142 356 357 358 359 143 437 438 439
CT3origin L 136 137 138 139
CT3proto L 137 138
L 138
CT3others L 356
CT7
11-10S3 L 144 145 146 147 148 149 150
CT7new+ L 528 144 145
CT7origin L 144
CT11
2010Env. L 222 223 224 225 226 227 228 229
CT11proto L 224 225 226
CT11others L 830 224 225
CT15
2010Env. L 273 274 275 276 · · 300
CT15new+ L 792 793 273
L 802 803 273
CT15olrigin L 273 274 275
CT16
2010Env. L 301 302 303 304 305 306 307 308 309 310 311
CT16new+ L 813 301 302
CT16isolate L 301 302 303 304
CT16proto L 302
L 305
CT18
2010Env. L 325 326 327 328 329 330 331 332 333 334 335 336 337
CT18new+ L 771 772 773
L 773 325 326
CT18origin L 325 326 327 328
CT18proto L 326
L 328
L 330 331 332
CT24
11-30E6 L 118 119 120 121 122 123 124 125 126 127 128 129 130 477 478 479 480 481 482 483 484 485 486 487 488 489 490
CT24origin L 118 119 120 121
CT24proto L 119
L 478 479 480
CT24others L 118 126 127
CT25
11-10E4 L 91 92 93 94 95 96 97 98 99 100 101 102 103
CT25origin L 91 92 93 94
CT25proto L 92
L 97 98 99 100
CT26
2010Env. L 104 105 106 201 202 203
CT26isolate L 104 105 106
CT26proto L 106 201 202 203
CT29
11-10S8 L 87 88 89 90 156 157 158 159 160 161 162 163 164 165 166 167 168 169 170 171 172
CT29new+ L 806 807 87 88
CT29isolate L 87 88 89 90
CT29proto L 172 596 165 800
CT33
11-30W55 L 107 108 109 · · 117 209 210 211 212 213 452 453 · · 473 474 475 476 214 215 216 217 218 219 220 221
CT33origin L 107
CT33proto L 211 212
CT33others L 616 617 215

Figure S3. Continued.

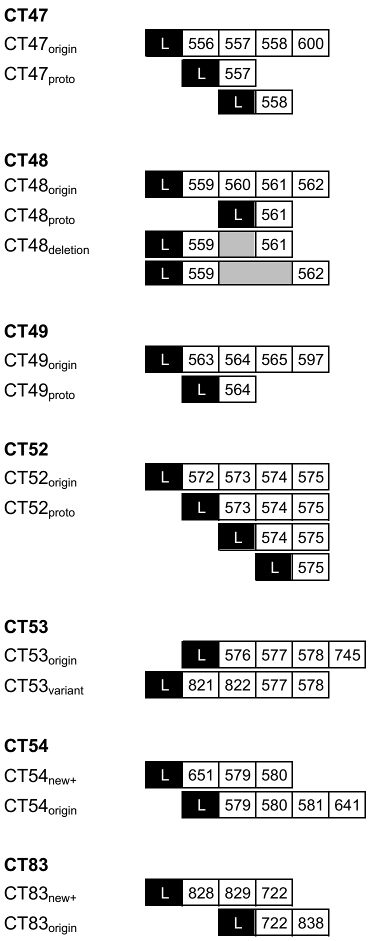

Figure S4.

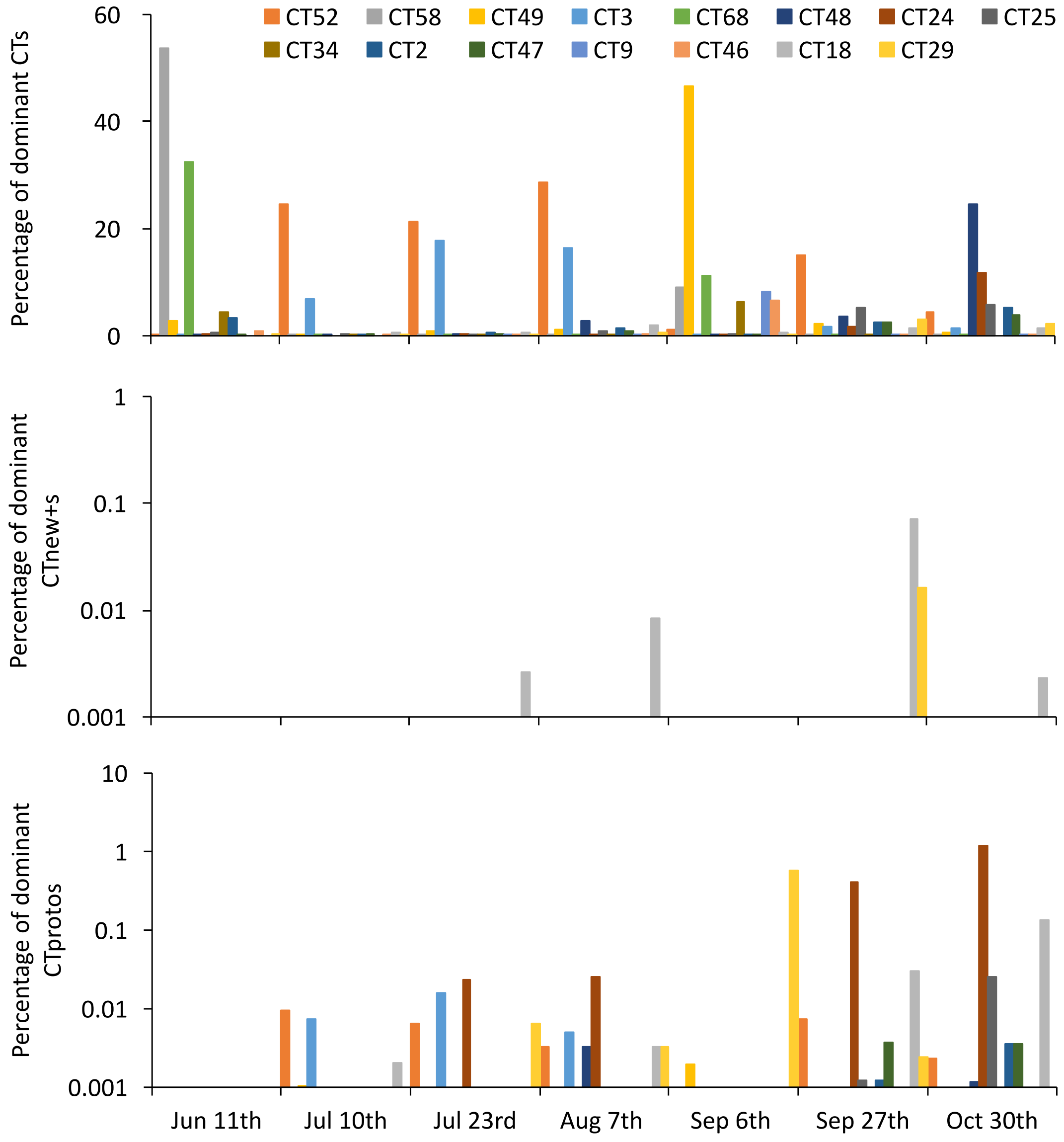
Percentage of dominant CTs
60
40
20
0
CT52
CT58
CT49
CT3
CT68
CT48
CT24
CT25
CT34
CT2
CT47
CT9
CT46
CT18
CT29
Percentage of dominant CTnew+s
1
0.1
0.01
0.001
Percentage of dominant CTprotos
10
1
0.1
0.01
0.001
Jun 11th
Jul 10th
Jul 23rd
Aug 7th
Sep 6th
Sep 27th
Oct 30th
